# Mindfulness, Life Skills, Resilience, and Emotional and Behavioral Problems for Gifted Low-Income Adolescents in China

**DOI:** 10.3389/fpsyg.2020.00594

**Published:** 2020-03-31

**Authors:** Chien-Chung Huang, Yafan Chen, Huiying Jin, Marci Stringham, Chuwei Liu, Cailee Oliver

**Affiliations:** Huamin Research Center, School of Social Work, Rutgers, The State University of New Jersey, New Brunswick, NJ, United States

**Keywords:** mindfulness, life skills, resilience, emotional and behavioral problems, gifted adolescents, China

## Abstract

In contrast to emotional and behavioral problems (EBPs), which can disrupt normal adolescent development, resilience can buffer the effects of stress and adverse childhood experiences and can help youth overcome adversity. While research has looked at the relationship between adolescent resilience and EBPs, current literature relatively lack a discussion of a strengths-based approach of resilience framework, nor discuss non-western sociocultural contexts. In this study, we utilized the resilience theory to examine the effects of individual mindfulness and life skills on resilience and consequently on EBPs in a group of low-income and gifted adolescents in China. A secondary data of 152 adolescents from a specialized school for low-income and gifted students in Guangzhou, China was used for the analysis. The findings from structural equation modeling indicated that mindfulness and life skills were associated with heightened resilience and reduced EBPs. In addition, resilience reduced EBPs for this group of adolescents. These findings underscore the promise of mindfulness and life skills training on increasing resilience and reducing EBPs in gifted adolescents.

## Introduction

Children from low-income families tend to have significantly higher emotional and behavioral problem (EBPs) than children from higher-income families (Baek and Yoo, 2017; Choi et al., 2019). Empirical studies have shown that adolescents with higher-than-average levels of EBPs are associated with a variety of developmental, social, and behavioral delays, as well as myriad mental and physical health diagnoses (Jokela et al., 2009; Slopen et al., 2013; Jones et al., 2015). Childhood EBPs are also associated with negative mental states such as anxiety, academic under-achievement, delinquency, and depression (Broidy et al., 2003; Bahmani et al., 2016). This link between low-income students and the proclivity for academic under-achievement and the associated EBPs can be particularly devastating for an adolescent in the Chinese school system (Lu et al., 2018; Chen et al., 2018). In China, those who come from lower socioeconomic status families often find that education is their primary way to improve their lives and obtain a well-paying job in the competitive Chinese labor market (Ye, 2015; Chen et al., 2018).

Gifted children can face some of the same challenges as children exhibiting EBPs. As defined by the United States Elementary and Secondary Education Act, gifted and talented students “…give evidence of high achievement capability in areas such as intellectual, creative, artistic, or leadership capacity, or in specific academic fields, and who need services and activities not ordinarily provided by the school in order to fully develop those capabilities” (Zipp, 2016). In an academic setting, high achievement capability is marked by rapid uptake of new information, complex/multi-faceted thought processing, and creative problem solving (Coleman and Cross, 2005). As a policy measure, many consider youth who perform in the top 10% in a given domain and in relation to a regional norm to be a likely indicator of those who would most benefit from specialized interventions, programs, and services (Coleman and Cross, 2005).

Although gifted students are exceptionally endowed with abilities that correspond to success in an academic setting, they are equally at risk with non-gifted peers of experiencing social and emotional difficulties that could impair their proper psychological development (Solow, 1995). Giftedness would seem to add a layer of complexity to their development, leading to some unique psychological issues (Renati et al., 2017). The set of emotional difficulties that prevail among the gifted population can include social isolation, perfectionism, anxiety, rejection, intolerance of criticism, and resistance to authority (Freeman, 1983; Gardynik and McDonald, 2010; Guenole et al., 2013). Gifted children might also experience unrealistic expectations from teachers and parents, high test anxiety, and difficulties adapting to their social environment (Eren et al., 2018). Some gifted children struggle with the speed at which their minds work compared with their emotional maturity, as well as the constancy of their constantly active mind (Tolan, 2018).

Many of these difficulties are compounded for gifted children born into poverty. It has been shown that, in the United States, elementary and middle schools with large numbers of low-income students lack the advanced programs in elementary-school settings required to prepare gifted students to engage in and succeed at definitive educational experiences in high school and early college (e.g., Algebra II-level math classes and beyond) that are determinative of high academic achievement in adulthood (Burney and Beilke, 2008). Low-income schools also often do not have the funds to train educators in how to work with gifted and talented students, which can lead to high-achieving students becoming bored with the subject material (Freeman, 1983; Haberline and O’Grady, 2018; Olszewski-Kubilius and Corwith, 2018).

The gifted-student population, although faced with a unique set of emotional challenges, are at similar risk to the broader adolescent cohort of experiencing negative outcomes as a result of EBPs (Chen et al., 2018). Despite the strong potential for EBPs to compromise a healthy developmental course, further research has shown that adult outcomes for youth strongly exhibiting these risk factors are widely varied (Sroufe et al., 2005; Masten, 2018). A factor that appears to mitigate the downside risk of problem behaviors in gifted youth as they develop into adults is their individual capacity for resilience (Chen et al., 2018).

Health professionals used to focus on interventions based on pathology where they fixed deficits or problems (Wagnild and Collins, 2009; Masten, 2014). However, a growing body of research emphasizing the protective effects of resilience has shifted health professionals’ focus from “fixing” deficits toward assessing an individual’s strengths and then developing an intervention built on that individual’s existing capabilities (Masten, 2014; Wagnild and Collins, 2009). Resilience is a dynamic mental process marked by the ability to engage in positive, adaptive behaviors that allow for successful emotional and social development in the face of significant adversity or stress (Wagnild and Young, 1993; Luthar, 2003; Rutter, 2012). Researchers are interested in resiliency because of its strong presence in individuals that exhibit adaptive functioning amidst life circumstances that put them at risk of developing maladaptive psychopathologies (Wagnild and Collins, 2009; Bethell et al., 2014; Masten, 2018).

The resiliency theory is a strengths-based conceptual framework of child and adolescent development (Rutter, 1993; Fergus and Zimmerman, 2005; Montero-Marin et al., 2015). It emphasizes on positive individual and contextual variables that interfere or disrupt developmental trajectories from risk to negative problems and outcomes. Individual variables are referred to factors that reside within individuals such as self-efficacy, while context variables refer to factors outside individuals such parental or neighborhood support. Resilience theory posits a conceptual framework on explaining how individual and contextual variables increase resilience and that resilience consequently and effectively cope with EBPs among individuals.

We focus on two individual variables that might increase resilience in this study: individual mindfulness and life skills. Mindfulness can be defined as purposely paying attention to the present moment and reacting in a non-judgmental fashion Kabat-Zinn (2003). The receptive and open state of awareness stemming from mindfulness can benefit both mental and physical health (Black and Fernando, 2014; Bluth and Eisenlohr-Moul, 2017). Many articles have linked mindfulness to positive effects on relationships, emotions, and behaviors (Keng et al., 2011; Edmonds et al., 2014; Mak et al., 2018). Cultivating mindfulness skills through targeted interventions can have positive effects on both adolescents’ and adults’ psychological adjustment (Bergin and Pakenham, 2016; Ramler et al., 2016; Cutright et al., 2019). Mindfulness can also lead to better self-regulation, which has been shown to be a protective factor for children from low socioeconomic statuses (Flouri et al., 2014). Gifted children, in particular, can feel additional stress because of the speed at which their minds work. Mindfulness helps individuals have better control over their own focus and attention. Gifted children can also spend a lot of time in their minds, and mindfulness can help the gifted child to slow down, and focus on their bodies and how they are responding to their environments (Tolan, 2018).

The World Health Organization (WHO) defines life skills as “abilities for adaptive and positive behavior that enable individuals to deal effectively with the demands and challenges of everyday life” (World Health Organization [WHO], 1997, p. 1). Life skills are made up of various interrelated components working together and reinforcing one another. These skills, essential in mitigating the constant challenges faced by growing adolescents, foster an individuals’ self-efficacy, self-esteem, psychosocial competence, and holistic self-development. Adolescents transitioning to adulthood face challenges which include pressure to abuse alcohol, abuse drugs, or participate in high-risk sexual behaviors (Botvin and Griffin, 2004; Nasheeda et al., 2018). Life skills allow individuals both to avoid these potentially damaging behaviors and to maximize other important protective factors (UNICEF, 2015). Life skills can be enhanced in an individual through well-designed interventions (Botvin et al., 1980). The Life Skills Training (LST) program effectively reduced behavioral problems in youth (Botvin et al., 1980; Botvin and Griffin, 2014; Velasco et al., 2017). The LST program has proven effective for youth from a variety of socioeconomic levels (Botvin and Griffin, 2004, 2014). In general, the LST program teaches skills which are closely linked to key factors that promote resilience and positive emotional outcomes in youth.

The empirical literature has provided ample evidence of supporting theoretical framework of the resilience theory that mindfulness and life skills improve individual mental awareness and behavioral skills that lead to increased level of resilience (e.g., Huppert and Johnson, 2010; Jha et al., 2017; Kummabutr et al., 2017; Botvin LifeSkills Training, 2018; Huang et al., 2019) and that high level of resilience is associated with low level of EBPs among children and adolescents (e.g., Ziaian et al., 2012; Arslan, 2016).

However, current research on above relations tends to be based on studies in western countries and relatively lack a discussion of a strengths-based approach of resilience framework. Focusing on gifted low-income adolescents in China, this study utilize the resilience theory and hypothesize that individual mindfulness and life skills increase level of resilience which in turn reduces EBPs of adolescents.

## Materials and Methods

### Data

The data came from a secondary data collected by Guangdong University of Foreign Studies in June 2019. The sample was from students at the G High School in Guangzhou, China. The G High School is China’s first private, no-cost institution that recruits approximately 160 low-income, gifted adolescents from across China each year. The school was established in 2002 by the Y Foundation to execute on the foundation chairman’s desire that no gifted student fail, from having been born into indigent circumstances, to reach his or her full academic potential. The school is tuition free and boasts a highly advanced curriculum. Since founding the school in 2002, The Y Foundation has invested upward of 260 million yuan in facilities, programs, and tuition costs.

Eligibility to apply to the G School is based on a review of family resources and demonstration of exceptional scores on high school entrance examinations: students’ scores need to be above the ones required by key county- or prefecture-level high schools, and the student’s family must fall below the poverty line for their locale. For students meeting the applicable baseline criteria, the G School administers its own written examination and conducts oral interviews from which to make its final admission decisions. Compared to the written examination that emphasizes on intellectual ability, the interview focuses on the comprehensive development of the applicants, including virtue and volunteering.

The study adhered the ethical standards and the research protocol was reviewed and approved by the Ethics Committee of a Chinese University. Among 160 first-year students, four students were not available to participate at the time of the survey, and four students provided incomplete survey results. The final number of students included in the study was 152.

### Measures

Emotional and behavior problems were measured with an abridged, adolescent version of the Self-Description Questionnaire (SDQ) (Marsh, 1990; Bendheim-Thoman Center for Research on Child Wellbeing, 2013). This 14 item version of the SDQ assesses both internalizing and externalizing behaviors. The SDQ Chinese version, which we used, has shown good validity, reliability, and cultural applicability among youth in China (Yeung and Lee, 1999; Marsh et al., 2000; Leung et al., 2016; Lu et al., 2018). This study’s Cronbach’s alpha for these 14 items was 0.81. Six questions measured externalizing behaviors such as: “It’s hard for me to finish my schoolwork,” “It’s hard for me to pay attention”; “I get distracted easily,” “I often argue with other kids,” and, “I get in trouble for talking and disturbing others.” The Cronbach’s alpha for this study on these six items was 0.66. Eight questions measured internalizing behaviors (those manifested in thoughts or feelings): “I often feel lonely,” “I feel ashamed when I make mistakes at school,” “I worry about doing well in school,” “I feel sad a lot of the time,” “I worry about finishing my work,” “I worry about taking tests,” “I worry about having someone to play with at school,” and “I feel angry when I have trouble learning.” Cronbach’s alpha on these items was 0.80 for this study. For each of the 14 items, participants rated the frequency of the noted experiences on a scale of 0–3 with descriptions ranging from “not at all true” to “very true.” Summary scores were computed by simple addition of the responses. Externalizing-behavior scores ranged from 0 to 18 and internalizing-behavior scores ranged from 0 to 24. The total SQD score ranged from 0 to 42. We used internalizing behaviors to measure emotional problems and utilized externalizing behaviors to gage behavioral problems. The total SDQ score was used for the level of EBPs.

Resilience was assessed using a concise form of the Resilience Scale (Wagnild and Young, 1993), the 14-item Resilience Scale instrument (RS-14; Wagnild, 2016) due to time and resource limitation. Resilience-related traits are assessed by the RS-14, including personal characteristics that mitigate the destructive effects of adverse life circumstances on proper psychological adjustment (Wagnild and Young, 1993; Wagnild, 2016). Examples of items in the RS-14 include: “When I’m in a difficult situation, I can usually find my way out of it,” “My belief in myself gets me through hard times,” and “I am determined.” RS-14 shows both cross-ethnic validity in the United States and good reliability amongst Chinese adolescents (Pritzker and Minter, 2014; Shi et al., 2016). For this study the Cronbach’s alpha was 0.90. Participants were instructed to rate each item according to how strongly they identified with each statement when considering themselves over the previous four weeks. Per-item scores ranged from 1 to 7 (strongly disagree to strongly agree). The sum of the item scores was computed and ranged from 14 to 98. Higher scores indicated a higher spot-measurement of resilience.

Two key variables assessed in the study were spot-measurements of life skills and mindfulness. The 14-item Mindful Attention Awareness Scale for Adolescents (MAAS-A) was used to measure mindfulness. The MAAS-A has been validated for adolescents from 14 to 18 years old (Brown et al., 2011) and both validity and reliability were shown for Chinese adolescents in the Chinese version. The 14 items asked participants to identify the frequency over the past 4 weeks with which they experience feelings, behaviors, or mindful thoughts such as “I rush through activities without being really attentive to them,” “I find myself doing things without paying attention,” or “I break or spill things because of carelessness, not paying attention, or thinking of something else.” The scale for each item ranged from 1 to 6 (almost never to almost always). In this scale higher scores indicated higher levels of mindfulness. The total of all scores provided ranged from 14 to 84, and the Cronbach’s alpha reached 0.80.

To measure life skills, a National Health Promotion Associates (2018) 10-item questionnaire was used. The instrument broke life skills into four sub-components: self-control, assertiveness, refusal and relaxation, and measured each of those. Participants were asked to evaluate, considering the past 4 weeks, how likely they were to exhibit the behaviors presented. Items used to assess refusal were: “Say no when someone tries to get you to drink beer, wine, or liquor,” “Say no when someone tries to get you to smoke marijuana or other drugs,” and “Say no when someone tries to get you to smoke a cigarette.” Items measuring assertiveness were: “Say no to someone who asks to borrow money from you,” “Tell someone to go to the end of the line if they try to cut in line ahead of you,” and “Tell someone if they give you less change (money) than you’re supposed to get back after you pay for something,” relaxation skills were highlighted with statements such as: “Breathe in slowly and deeply when faced with feelings of anxiety or nervousness” and “Relax all the muscles in your body, starting with your feet and legs.” And finally, items measuring self-control were: “You stick to what you are doing until you’re finished with it” and “If you find that something is really difficult, you get frustrated and quit.” Reverse coding was applied to all items except one so that higher scores would reflect a greater measure of life skills. The Cronbach’s alpha of the scale was 0.54. The total sum of item scores was used as our measure of life skills and ranged from 10 to 50. In addition to the preceding measures, we also included participant age and gender as the covariates for resilience (Huang et al., 2019).

### Analytic Strategy and Model

**FIGURE 1:**
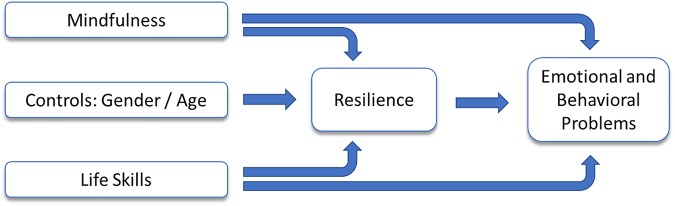
Path diagram of hypothesized model.

A descriptive analysis was performed to examine the distribution of each the main variables. Pearson correlation analysis was undertaken to observe the association between all variables. To further sort out the explanatory power of mindfulness and life skills on resilience as mediator of EBPs, we conducted structural equation modeling (SEM) to examine the effects of mindfulness and life skills on both resilience and EBPs. Regression techniques and SEM differ in that SEM allows not only examination of direct effects but also simultaneous analysis of indirect effects through mediating variables. A path model that depicts the relationships between mindfulness, life skills, resilience, and emotional and behavior problems was produced. The hypothesized model, as shown in Figure 1, posited that mindfulness and life skills affect EBPs of adolescents both directly by inculcating coping techniques and indirectly through their positive effects on resilience. Resilience is posited to directly reduce EBPs in adolescents. Age and gender, exogenous to the model and employed as controls, are modeled only to explain resilience. We hypothesize resilience fully mediate the effects of age and gender on EBPs. To evaluate model fit, several commonly used fit indices were used, including the comparative fit index (CFI), root mean square error of approximation (RMSEA), and the Chi-square test. STATA software 16.0 was used for all the analyses.

## Results

The sample’s descriptive statistics can be seen in Table 1. The average age of the 152 adolescents was 16.2 and 63.2% of them were male. On a scale from 14 to 84, where the standard deviation was 91, the average mindfulness score was 59.5. The average score was 40.1 for life skills on a scale of 10–50 where the standard deviation was 3.7. On a scale of 7–98, where the standard deviation was 11.9, the average resilience score was 72.8. The average SDQ score was 13.4 out of 42, with an average of 3.7 out of 18 for behavioral problems and 9.7 out of 24 for emotional problems.

**TABLE 1:** Descriptive statistics of sample characteristics.

***N* = 152**	**Mean or percentage (SD)**
Age	16.2 (0.6)
Gender [%]	
Male	63.2
Female	36.8
Mindfulness (14–84)	59.5 (9.1)
Life skills (10–50)	40.1 (3.7)
Resilience (7–98)	72.8 (11.9)
Emotional and behavioral problems, SDQ (0–42)	13.4 (6.1)
Emotional problems (0–24)	9.7 (4.7)
Behavioral problems (0–18)	3.7 (2.4)

*Figures in table are means or percentages, with standard deviations in parentheses.*

The Pearson correlation analyses of variables is shown in Table 2. The SDQ level showed significant correlation with resilience (*r* = −0.51, *p* < 0.001), mindfulness (*r* = −0.51, *p* < 0.001), life skills (*r* = −0.34, *p* < 0.001), and female (*r* = 0.19, *p* < 0.05). Further analyses, upon the request, showed that the correlation coefficients between female and behavioral and emotional problems, separately, were 0.03 (*p* > 0.05) and 0.23 (*p* < 0.01). Resilience levels significantly correlated with mindfulness (*r* = 0.46, *p* < 0.001), and life skills (*r* = 0.34, *p* < 0.001). Level of mindfulness was significantly correlated with level of life skills (*r* = 0.26, *p* < 0.01). The findings of the correlation analyses were in line with the hypothesized model.

**TABLE 2:** Pearson’s correlation coefficients of variables.

	**1**	**2**	**3**	**4**	**5**	**6**
1. SDQ	–					
2. Resilience	−0.51***	–				
3. Mindfulness	−0.51***	0.46***	–			
4. Life skills	−0.34***	0.34***	0.26**	–		
5. Age	0.09	−0.08	−0.03	0.01	–	
6. Female (male = 0)	0.19*	−0.15	−0.11	−0.11	−0.15	–

*N* = 152. *p < 0.05, **p < 0.01, ***p < 0.001.

**FIGURE 2:**
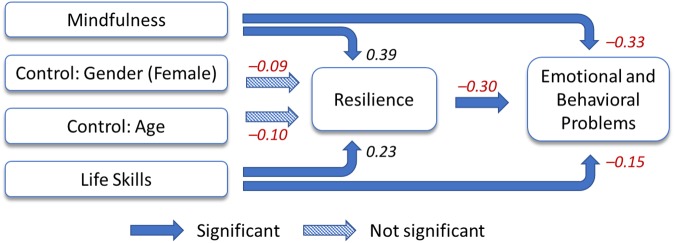
Estimated standardized coefficients of the path model.

Structural equation modeling was then used to examine the hypothesized model. Model-fit indices show that the observed data correspond well to the model. The Chi-square value was 3.48 (*p* = 0.18), RMSEA was 0.07, and CFI was 0.98. Figure 2 presents the standardized coefficients of the model, estimated by SEM, with bolded lines indicating the coefficients significant at *p* < 0.05 level and dotted lines indicating no statistical significance. The results indicated that resilience partially mediated the effects of mindfulness and life skills on EBPs. Specifically, mindfulness (β = 0.39, *p* < 0.001) and life skills (β = 0.23, *p* < 0.01) were directly associated with high resilience. Resilience was directly and negatively associated with the score of EBPs (β = −0.30, *p* < 0.001). Mindfulness (β = −0.33, *p* < 0.001) and life skills (β = 0.15, *p* < 0.05) were also directly associated with reduced EBPs. The estimated coefficients for gender and age on level of resilience were not significant in this sample.

Table 3 decomposes the standardized effects on both adolescent EBPs and resilience. Both indirect effects of mindfulness (β = −0.12, *p* < 0.01) and life skills (β = −0.07, *p* < 0.05) were significant on EBPs, a finding consistent with resilience having mediated the effects of mindfulness and life skills on EBPs. The total effects life skills and of mindfulness on EBPs were −0.45 (*p* < 0.001) and −0.22 (*p* < 0.001), respectively. These results provide evidence that resilience partially mediates the positive effects of mindfulness and life skills on emotional and behavior problems in adolescents. Mindfulness and life skills reduce emotional and behavior problems directly as well as indirectly through their influence on resilience.

**TABLE 3:** Decomposition of the standardized effects of emotional and behavioral problems (EBPs) of adolescents.

**Predictor**	**Dependent variable**	**Direct effect**	**Indirect effect**	**Total effect**
Mindfulness	Resilience	0.39***	−	0.39***
	EBPs	−0.33***	−0.12***	−0.45***
Life skills	Resilience	0.23***	−	0.23**
	EBPs	−0.15*	−0.07*	−0.22**
Female (male = 0)	Resilience	–0.10	−	–0.10
	EBPs	−	0.03	0.03
Age	Resilience	–0.09	−	–0.09
	EBPs	−	0.03	0.03
Resilience	EBPs	−0.30***	−	−0.30***

*N* = 152. *p < 0.05, **p < 0.01, ***p < 0.001.

We further examined the above model by separate SDQ subscales for emotional problems and behavioral problems, as shown in Tables 4, 5, respectively. The findings are generally in line with the conjoined finding reported above. There were, however, some notable differences. With respect to emotional problems as presented in Table 4, the above hypothesized model did not fit the data well without controlling for gender. In this case, the direct effect of trait female on emotional problems was strong (β = 0.15, *p* < 0.05). Accordingly, the total effect of female on emotional problems was significant (β = 0.18, *p* < 0.05), indicating female adolescents experienced relatively higher levels of emotional problems. The hypothesized model shows good explanatory power on behavioral problems in isolation as well. In this case, there was no direct effect of female on behavioral problems. However, the direct effect of mindfulness on behavior problems (β = −0.42, *p* < 0.001) was larger than that found for conjoined emotional and behavior problems presented in Table 3, and the direct effect of life skills on behavioral problems became small and insignificant (β = −0.05, *p* > 0.05).

**TABLE 4:** Decomposition of the standardized effects of emotional problems of adolescents.

**Predictor**	**Dependent variable**	**Direct effect**	**Indirect effect**	**Total effect**
Mindfulness	Resilience	0.39***	−	0.39***
	Emotional problems	−0.21**	−0.10***	−0.31***
Life skills	Resilience	0.23***	0	0.23**
	Emotional problems	−0.15*	−0.06*	−0.21**
Female (male = 0)	Resilience	–0.10	−	–0.10
	Emotional problems	0.15*	0.03	0.18*
Age	Resilience	–0.09	−	–0.09
	Emotional problems	−	0.02	0.02
Resilience	Emotional problems	−0.26**	−	−0.26**

*N* = 152. *p < 0.05, **p < 0.01, ***p < 0.001.

**TABLE 5:** Decomposition of the standardized effects of behavioral problems of adolescents.

**Predictor**	**Dependent variable**	**Direct effect**	**Indirect effect**	**Total effect**
Mindfulness	Resilience	0.39***	−	0.39***
	Behavioral problems	−0.42***	−0.09*	−0.51***
Life skills	Resilience	0.23***	−	0.23**
	Behavioral problems	–0.08	−0.05*	−0.13*
Female (male = 0)	Resilience	–0.10	−	–0.10
	Behavioral problems	−	0.02	0.02
Age	Resilience	–0.09	−	–0.09
	Behavioral problems	−	0.02	0.02
Resilience	Behavioral problems	−0.22**	−	−0.22**

*N* = 152. *p < 0.05, **p < 0.01, ***p < 0.001.

## Discussion

Our results support the resilience theory and indicate that individual mindfulness and life skills have strong direct and indirect effects on reducing EBPs for a group of low-income and gifted adolescents in China, with indirect effects arising through their positive effects on individual resilience; and all three variables are shown to have direct effects on reducing EBPs. The total positive effect of mindfulness on the presence of EBPs is larger than the effects of life skills and resilience.

Mindfulness practice can be shown to produce heightened attention. Its positive effect, then, is produced when individuals use enhanced focused attention to deliberately turn their attention toward sensory awareness in the present moment. Negative self-evaluation, often linked to negative internalizing behaviors, can thus be mediated by this present-moment, non-judgmental awareness. Distress tolerance also increases with mindfulness practice (Farb et al., 2012). Concurrently, mindfulness may induce a relaxed state and different responses, even when individuals fully experience the emotions arising from distressing stimuli (Hölzel et al., 2011). Accordingly, adolescents with relatively strong abilities in mindfulness will be able to reduce their automatic responses to, or divert their attention from, stressors that can lead to their exhibiting negative internalizing and externalizing behaviors.

Life skills also show strong overall effects on the incidence of EBPs, as well as reducing emotional problems in isolation. Although the direct effect of life skills on behavioral problems was not statistically significant, its indirect effect through resilience on behavioral problems was significant. The insignificance of the direct effect of life skills on behavioral problems may be an area of future research interest. This finding may result from the unique life circumstances of the present sample: students are housed in a boarding school environment under a semi-military disciplinary regime. It is conceivable, then, that the externalizing behavioral problems were low as a result of the environment and the observed effect (or lack thereof) might not be because of an inherent lack of association with the four life skills components: self-control, refusal, relaxation, and assertiveness.

It is evident that both mindfulness and life skills in this sample of gifted and low-income adolescents significantly and directly increased their resilience, a finding that potentially lessens the effects of adverse childhood experiences on later development through positive adaptation (Bethell et al., 2014). Together with the finding that resilience has strong, direct effects on the occurrence of EBPs, our findings point to a potential positive effect on development of an intervention for gifted adolescents that is shown to develop stronger mindfulness and life skills components. Such an intervention would be expected to strengthen their ability to face daily challenges by increasing individual resilience. All three factors would be expected to contribute to lower EBPs.

This study finds that female gifted adolescents exhibit higher levels of emotional problems than the male gifted adolescents in this sample. Chinese school-aged children are expected to have academic success and any school failures can bring shame and disgrace upon the family. Indeed, children who show EBPs through heightened internalizing symptoms are perceived as problematic and can face tremendous stigma from teachers and peers (Fu et al., 2016). Given the high pressure of upcoming college-entrance examinations and the uniquely strong imperative felt by gifted low-income students to succeed (Chan, 2003; Burney and Beilke, 2008), these findings indicate the urgent need for intervention and services for female students in this sample and beyond.

This study included several limitations that warrant further investigation. First, the data originated from a sample of 152 adolescents from one school who were chosen by a single, invariant set of admission criteria. Utilizing a larger and more representative sample is warranted in future research. Second, although we hypothesize a causative relationship, our results based on cross-section data only establish associations not necessarily causations: we propose that life skills and mindfulness, related to an individuals’ ability and awareness, positively affects their resilience and exhibition of EBPs. However, it is also possible that the relationship between these skills and EBPs will be shown to be bi-directional. Further longitudinal research to establish causation is thus necessary. Third, all information was self-reported by the students and therefore subject to intended and unintended reporting errors. Future study can consider triangulation of data collection, including sources from teachers, friends, and parents to valid the data. Fourth, life skill scale had a low Cronbach’s alpha, 0.54. Further study is warrant to investigate the extent of the scale items in Chinese culture. Fifth, although all instruments were from reliable scales, common method bias was not tested due to resource limitation. Further study can examine other forms of instruments to test the common method bias. Finally, future studies, combining both life skill training and mindfulness components in their interventions, could be employed to better explore the synergistic effect of these two field’s core concepts on adolescent well-being. In spite of these limitations, this study ranks among the first to link the path association among life skills, resilience, mindfulness, and EBPs in gifted, low-income adolescents.

## Data Availability Statement

The datasets generated for this study are available on request to the corresponding author.

## Ethics Statement

The studies involving human participants were reviewed and approved by the Review Committee, School of Public Administration, Guangdong University of Foreign Studies. Written informed consent from the participants’ legal guardian/next of kin was not required to participate in this study in accordance with the national legislation and the institutional requirements.

## Author Contributions

C-CH took full responsibility for leading the research, data analysis, and writing the sections “Methods” and “Results.” YC took full responsibility for the literature review and publication process, and shared responsibility for the section “Discussion” with HJ, CL, and CO. HJ took full responsibility for the section “Introduction.” MS took full responsibility for manuscript revision.

## Conflict of Interest

The authors declare that the research was conducted in the absence of any commercial or financial relationships that could be construed as a potential conflict of interest.
